# Clinical application of immunogenic cell death inducers in cancer immunotherapy: turning cold tumors hot

**DOI:** 10.3389/fcell.2024.1363121

**Published:** 2024-05-07

**Authors:** Yiman Han, Xin Tian, Jiaqi Zhai, Zhenyong Zhang

**Affiliations:** Department of Oncology, Shengjing Hospital of China Medical University, Shenyang, Liaoning, China

**Keywords:** immunogenic cell death (ICD), tumor microenvironment (TME), cancer immunotherapy, immunosuppressive tumors, inducers, clinical application

## Abstract

Immunotherapy has emerged as a promising cancer treatment option in recent years. In immune “hot” tumors, characterized by abundant immune cell infiltration, immunotherapy can improve patients’ prognosis by activating the function of immune cells. By contrast, immune “cold” tumors are often less sensitive to immunotherapy owing to low immunogenicity of tumor cells, an immune inhibitory tumor microenvironment, and a series of immune-escape mechanisms. Immunogenic cell death (ICD) is a promising cellular process to facilitate the transformation of immune “cold” tumors to immune “hot” tumors by eliciting innate and adaptive immune responses through the release of (or exposure to) damage-related molecular patterns. Accumulating evidence suggests that various traditional therapies can induce ICD, including chemotherapy, targeted therapy, radiotherapy, and photodynamic therapy. In this review, we summarize the biological mechanisms and hallmarks of ICD and introduce some newly discovered and technologically innovative inducers that activate the immune system at the molecular level. Furthermore, we also discuss the clinical applications of combing ICD inducers with cancer immunotherapy. This review will provide valuable insights into the future development of ICD-related combination therapeutics and potential management for “cold” tumors.

## 1 Introduction

Immunotherapy has emerged as a promising treatment option in recent years in certain types of cancers. It is a regimen that treats cancer by regulating the body’s immune system, using immune checkpoint inhibitors (ICIs), monoclonal antibodies, T-cell transfer therapy, and tumor vaccines. The clinical application of immunotherapy has considerably improved the prognosis of multiple types of tumors, in turn improving the prognosis of many patients. However, when it comes to immunosuppressive tumors that are more resistant to immunotherapy, some challenges still urgently need to be addressed. Firstly, patients with “cold” tumor, characterized by an immunosuppressive tumor microenvironment (TME), insufficient T cell infiltration, or a lower immunoscore, gain few benefits from immunotherapy (Vonderheide, 2018; Galon and Bruni, 2019; Zhang and Zhang, 2020).” In addition, malignant cells can evolve several immune-escape mechanisms to prevent being monitored and eliminated (Hanahan and Weinberg, 2011), making immunotherapy more challenging. However, a cellular process discovered in the recent decades called immunogenic cell death (ICD) has highlighted promising tumor management methods (Li C. et al., 2022). In general, ICD remodels the original immunosuppressive TME via the release of various damage associated molecular patterns (DAMPs) and stimulates an immune response to suppress tumor growth, converting the “cold” tumor into a “hot” phenotype. This increases the efficacy of immunotherapy (Wang-Bishop et al., 2020) in patients who had minimal benefited from initial clinical treatment. Importantly, extensive studies have shown that traditional therapies, such as chemotherapy (Fabian et al., 2021; Kodumudi et al., 2010), targeted therapy, radiotherapy (RT) (Huang et al., 2021; Vaes et al., 2021), and photodynamic therapy (PDT) (Yu et al., 2023) can function as ICD inducers to activate the host-reactive immune response (Rodriguez-Ruiz et al., 2020; Zhu et al., 2021), although they were originally acknowledged to directly kill tumor cells (Cherniavsky et al., 2015). Notably, only a limited number of these traditional regimens serve as ICD inducers or induce sufficient ICD (Fu et al., 2021). Thus, investigators need to identify novel inducers (Sen et al., 2023) and explain the rationale behind various combination approaches to promote the efficacy of immunotherapy.

In this review, we briefly summarize the key hallmarks of ICD, discuss their implications for the host immune system, introduce novel regimens for eliciting ICD, and analyze their potential clinical applications.

## 2 Biology of ICD

### 2.1 Definition and antitumor effects of ICD

ICD is defined as “a form of regulated cell death that is sufficient to activate an adaptive immune response in immunocompetent hosts” (Galluzzi et al., 2018). Unlike necrosis or apoptosis, ICD is characterized by diverse molecular hallmarks and a subsequent antigen-specific immune response (Medrano et al., 2017). It can improve the maturation of dendritic cells (DCs), attract mature DCs to gather around dying tumor cells (Kobayashi and Choyke, 2019), and stimulate their antigen cross-presenting ability to activate cytotoxic T lymphocytes offering prolonged adaptive immunity (Azizi et al., 2023; Wei et al., 2021). In addition to the increase in cytotoxic T lymphocyte function, their infiltration is also enhanced (Wei et al., 2021). Simultaneously, there is a significant decrease in the proportion of suppressor cells, such as regulatory T lymphocytes (Li et al., 2021).

### 2.2 Necessary conditions for ICD

**FIGURE 1:**
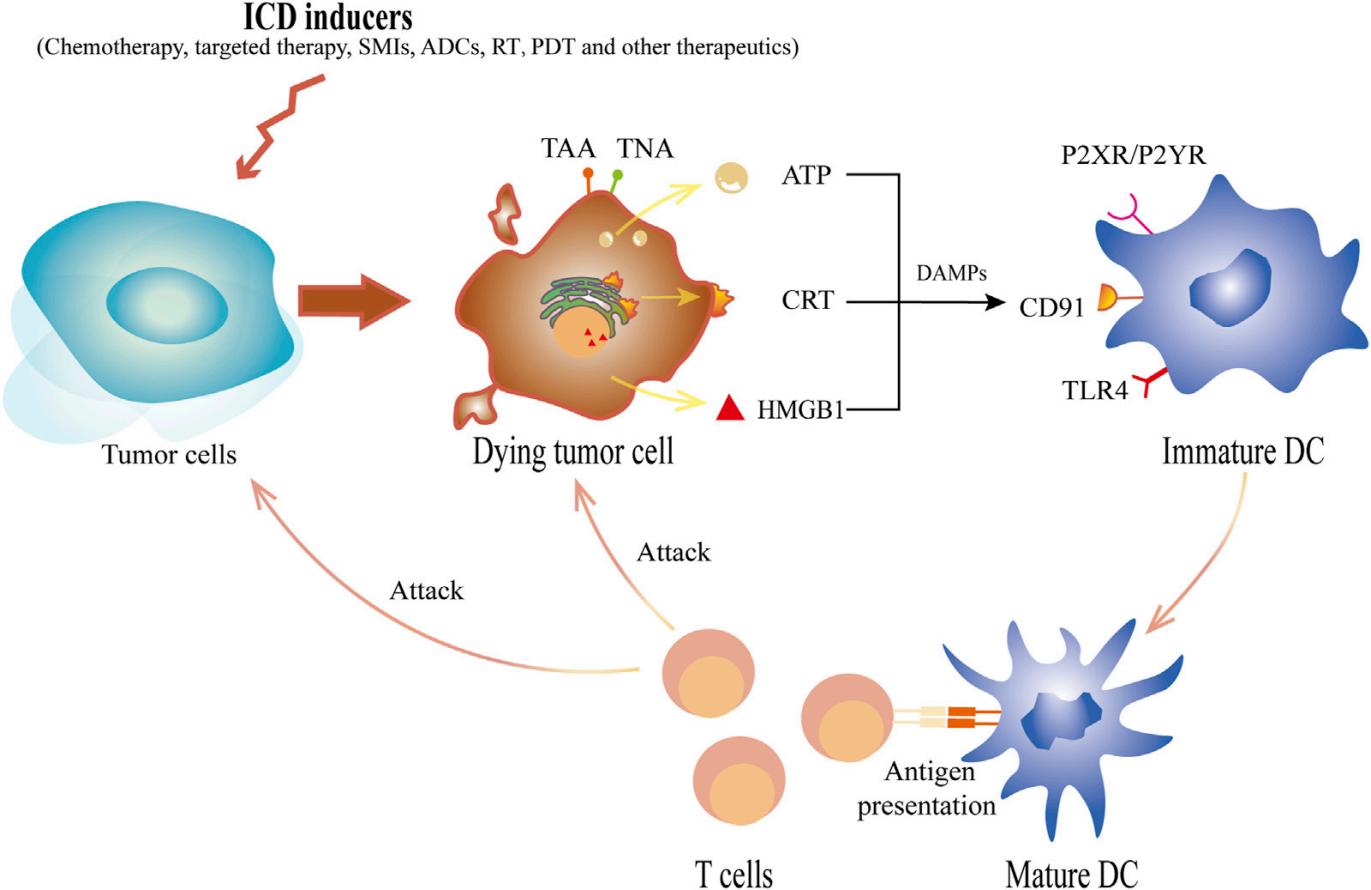
The schematic diagram of ICD inducers acting on tumor cells to induce ICD. ICD inducers, including chemotherapy, targeted therapy, SMIs, ADCs, RT, PDT and other therapeutics, cause tumor cells to die and express tumor antigens, TNAs or TAAs, on the cell surface. Concurrently, DAMPs released by tumor cells undergoing ICD can selectively bind to different specific receptors on DCs. In the early stage of ICD, ATP, one of the major player secreted by neoplastic cells, acts on the purinergic receptor P2XR and P2YR from DCs. Additionally, the translocation of CRT to the surface, which will be recognized by CD91, sends a “eat me” signal. Released in the late stage, HMGB1 binds with TLR-4 and initiates antigen presentation. Ultimately, these DAMPs initiate a cascade of antitumor reactions that lead to the recruitment, maturation and antigen presenting stimulation of DCs, thus eventually causing the polarization of T cells as well as in turn killing dying cells and other tumor cells. ICD, immunogenic cell death; RT, radiotherapy; TNAs, tumor neoantigens; TAAs, tumor-associated antigens; DAMPs, damage associated molecule patterns; DCs, dendritic cells; ATP, adenosine triphosphate; SMIs, small-molecule inhibitors; ADCs, antibody-drug conjugates; PDT, photodynamic therapy; CRT, calreticulin; HMGB1, high-mobility group protein box 1; TLR-4, Toll-like receptor 4.

**FIGURE 2:**
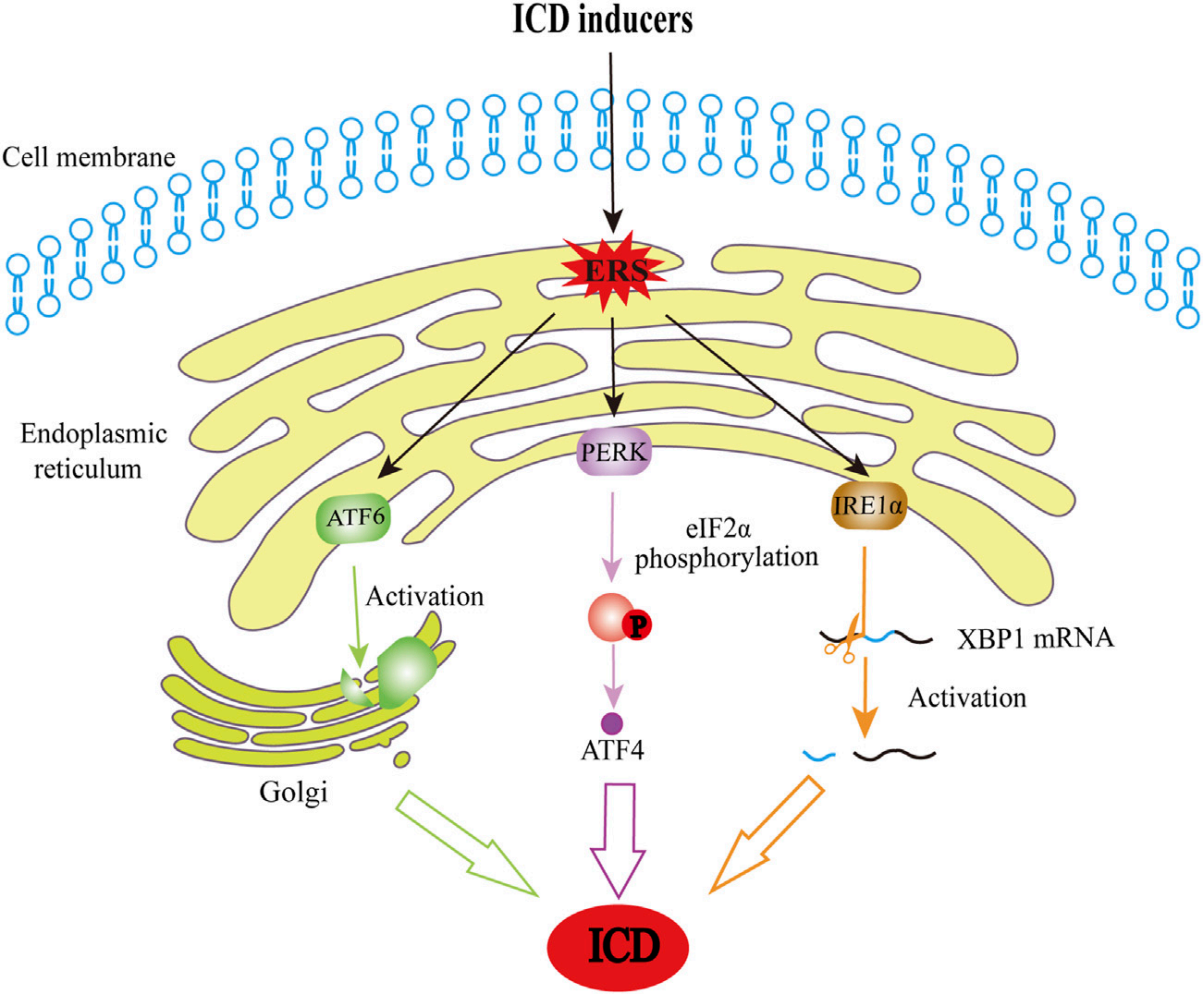
The schematic diagram of ERS-induced cell death. When affected by ICD inducers, cells are exposed to stresses that disturb protein folding and therefore lead to the production of ERS, a process that is also a critical step in ICD induction. In this regard, ERS activates IRE1α, and it plays a role with its endonuclease activity in splicing and activating XBP1 mRNA. PERK activation can induce phosphorylation of eIF2α, and phosphorylated eIF2α allows for selective translation of ATF4. Moreover, ATF6 will move to the Golgi apparatus and be cleaved into active fragments. These pathways constitute the main pathways through which ERS results in ICD. ICD, immunogenic cell death; ERS, endoplasmic reticulum stress; IRE1α, inositol-requiring kinase 1; XBP1, X-box-binding protein 1; PERK, protein kinase R-like ER kinase; eIF2α, eukaryotic initiation factor-2α; ATF4, activating transcription factor 4; ATF6, activating transcription factor 6.

In general, the ICD immunogenic properties manifest in two aspects: antigenicity and adjuvanticity (Kroemer et al., 2022). When undergoing cell death, cancer cells must have sufficient antigenicity to trigger an adaptive immune response, resulting in ICD. Tumor neoantigens (TNAs) are a source of antigenicity, arising from accumulated mutations acquired as tumors evolve (Vitale et al., 2019). Additionally, antitumor immune responses can be mediated by tumor-associated antigens (TAAs) (Sprooten et al., 2019) that are not specific to neoplastic cells. The adjuvant aspects of ICD refer to the process by which antigen-presenting cells efficiently present antigens to T cells in the presence of co-stimulatory molecules (Jhunjhunwala et al., 2021). ICD is triggered by endoplasmic reticulum stress (ERS) and/or reactive oxygen species (ROS)-induced stress. It is intricately linked to the molecular biomarkers represented by DAMPs released during cell death, such as calreticulin (CRT), high-mobility group protein box 1 (HMGB1), adenosine triphosphate (ATP), family of heat shock proteins (HSP), cellular nucleic acids such as SAP130, annexin A1 (ANXA1), and immunostimulatory and chemotactic cytokines such as IFN-I, CCL2, CXCL1, and CXCL10 (Apetoh et al., 2007; Sistigu et al., 2014). Different ICD inducers cause cells to release different DAMPs, each of which plays an antitumor role with its unique mechanisms, as elucidated by many previous preclinical and clinical studies (Figure 1). Mechanistically, these inducers can be divided into two major categories based on whether they act directly on the endoplasmic reticulum (ER) (Krysko et al., 2012), Type I ICD inducers (which target is unrelated to the ER, the secondary ERS that it causes tends to be milder) and Type II inducers (which directly act on the ER, and cause relatively potent ERS). When the stress exceeds the adjustable range, it activates inositol-requiring enzyme 1 alpha (IRE1α), and the endonuclease activity at the end of IRE1α specifically splices the mRNA of the transcription factor X-box-Binding Protein 1 (*XBP1*), which, in turn, activates *XBP1* mRNA. Additionally, activated protein kinase RNA-like endoplasmic reticulum kinase (PERK) induces the phosphorylation of eukaryotic initiation factor-2α (eIF2α), which allows for selective translation of activating transcription factor 4 (ATF4) and inhibits the synthesis of general proteins. The production of ERS also activates the membrane protein-activating transcription factor 6 (ATF6), which is transferred to the Golgi apparatus and cleaved into active fragments. Eventually, these three pathways can elicit the transcription of related genes in the nucleus, and ultimately cause translocation of DAMPs and ICD. The related pathways are shown in Figure 2. Of note, most current known inducers, such as chemotherapy and RT, are type I (Sen et al., 2022) and their immune efficacy needs to be enhanced by new biomaterials or combination therapies.

### 2.3 Novel DAMPs

In addition to commonly known molecular patterns, a new type of immunoactive polypeptide, proTα, known as thymosin, is gradually being confirmed as a new member of the DAMP family and plays a key role in many aspects such as diagnosis, treatment, and prognosis (Birmpilis et al., 2022). Notably, not all molecular markers elicited by dying malignant cells play a role in pathways that inhibit tumor progression. Molecules that inhibit immunity also constitute the inhibitory TME. For example, prostaglandin E2 (PGE2) is an inhibitory DAMP that counteracts the function of immunostimulatory DAMPs (Hangai et al., 2016). Gemcitabine elicits an immunostimulatory DAMP release; however, it also triggers PGE2 release; therefore, a PGE2 blockade is needed to tip the balance between the two types of DAMPs and help induce ICD (Hayashi et al., 2020). Similarly, adenosine (ADO) is an immunosuppressive DAMP (Stultz and Fong, 2021). While, CD39 and CD73 are extracellular nucleotidases that can precisely regulate the content of ATP and ADO in the purinergic pathway of the tumor cell immune microenvironment and maintain the balance between antitumor immunity and immunosuppression (Bao and Xie, 2022).

By detecting DAMPs, researchers can gain a clearer understanding of the mechanisms underlying ICD and determine whether a drug can play an antitumor role by inducing ICD. Detection strategies for different DAMPs were described in previous reviews (Fucikova et al., 2020; Galluzzi et al., 2020). The detection of ICD hallmarks is critical to determine whether a cell death process is immunogenic or typical of apoptosis. For example, transgenic CD8^+^ T cells and natural killer cells co-cultured with tumor cells cause cytotoxic tumor cell death. The observation of ICD markers demonstrates that cytotoxic cell death is immunogenic (Minute et al., 2020). Additionally, the main components of ICD are of great use in identifying new drugs with immunogenic properties for potential clinical applications. Thus, DAMP signaling may be essential to select patient-specific chemotherapy and tumor-specific therapeutic strategies (Radogna et al., 2019). Notably, not all drugs are qualified inducers although some agents can cause potent intracellular stress and alter the relevant molecular markers (Kroemer et al., 2022; Liu X. et al., 2023).

## 3 ICD inducers and molecular mechanism

### 3.1 Chemotherapy

Traditional chemotherapeutic agents with immunogenic effects summarized in the literature include (but are not limited to) anthracyclines (such as doxorubicin (DOX), epirubicin, mitoxantrone, and idarubicin), platinum-based drugs (such as cisplatin and oxaliplatin), anti-microtubule drugs (such as paclitaxel and docetaxel), bortezomib, cyclophosphamide, 5-fluorouracil, and so on (Gmeiner, 2020; Marinello et al., 2018; Shi et al., 2023; Wu and Waxman, 2018; Zhai et al., 2023) (Table 1). Here, we introduce innovations and continuous modifications based on these identified drugs, as well as discoveries of existing agent functions.

**TABLE 1:** ICD inducers that have been published before.

Type	Site of action	Mechanism	Immunogenic role	Ref
Chemotherapy				
Anthracyclines (DOX, EPB, MIT and IDA)	DNA topoisomerase II	Caspase activation	Promote the production of IFN and stimulate autophagy	Marinello et al. (2018)
		Activation of cGAS/STING pathway		
Platinum drug (DDP, OXA)	DNA	Ribosome biogenesis stress	Induce expression of danger signals and trigger T cell-mediated immune responses	Shi et al. (2023)
				Zhai et al. (2023)
Anti-microtubule drugs (PTX, DTX)	Microtubules	Mitotic arrest of cell circle and ERS	Induce the translocation of CRT and ERp57 and upregulate the immune cells infiltration while downregulate suppressor cells	Shi et al. (2023)
Bortezomib	26S proteasome inhibitor	Activation of cGAS/STING pathway	Trigger maturation of DCs and improve T-cell activation	Zhai et al. (2023)
Cyclophosphamide	DNA	Cytotoxic damage and induction of ICD	Improve phagocytosis of DCs, enhance infiltrating lymphocytes and T-cell activation	Wu and Waxman (2018)
5-fluorouracil	Thymidylate synthase	Induction of ICD when combined with other drugs	Improve phagocytosis of DCs and trigger immune responses mediated by T cells	Gmeiner. (2020)
Targeted therapy				
Cetuximab	EGFR	Interference of EGFR-ligand interaction and signaling inhibition and induction of ICD in combination with chemotherapy	Improve phagocytosis of DCs Enhance immune cell infiltration into metastatic sites	Inoue et al. (2017), Pozzi et al. (2016)
Ibrutinib	Bruton’s tyrosine kinase	Irreversible inhibition of Bruton’s tyrosine kinase and promotion of ICD	Stimulate antigen-presenting cells	Galluzzi et al. (2015), Sagiv-Barfi et al. (2015)
Tyrosine kinase inhibitors (crizotinib, foretinib, canertinib, lapatinib, lestaurtinib, and ceritinib)	Tyrosine kinase	On-target effect: inhibition of ALK, ROS1 or MET	Enhance T lymphocytes infiltration and elicit a memory immune response	Xu et al. (2022)
		Off-target effect: when combined with cisplatin		
Cyclin-dependent kinase inhibitors (abemaciclib and palbociclib)	CDK	Induction of cell circle arrest and apoptosis	Increase MHC-1 cells significantly	Schoenwaelder et al. (2021)
Radiotherapy				
RT	DNA	Free radicals (such as ROS)	Stimulate antigen-presenting cells and T-cell activation	Galassi et al. (2023), Hannon et al. (2023)

DOX, doxorubicin; EPB, epirubicin; MIT, mitoxantrone; IDA, idarubicin; DDP, cisplatin; OXA, oxaliplatin; PTX, paclitaxel; DTX, Docetaxel; IFN, interferon; ERS, endoplasmic reticulum stress; CRT, calreticulin; ERp57, protein disulfide isomerase A3; DCs, dendritic cells; ICD, immunogenic cell death; EGFR, epidermal growth factor receptor; CDK, cyclin-dependent kinase; MHC-1, major histocompatibility complex class 1; RT, radiotherapy; ROS, reactive oxygen species.

#### 3.1.1 Chemotherapy combination complex

The relatively low ICD intensity induced by some of the earliest chemical inducers prompted researchers to compensate for this shortcoming through technological improvements. For example, to enhance the immune regulatory function of platinum-based drugs through structural improvements. Kostrhunova et al. (2023) synthesized a series of platinum (IV) derivatives based on the platinum (II) complex, [PtII56MeSS], which contained diclofenac axial ligands, and compared their outcomes to those of cisplatin. This new platinum-based complex led to platinum-induced ICD and possessed the characteristics of diclofenac, with the presence of diclofenac increasing the selectivity and cytotoxicity of platinum on six human cancer cell line (cervical carcinoma HeLa, breast adenocarcinomas MDA-MB-231 and MCF-7, colon carcinoma HCT-116, ovarian carcinoma A2780 and A2780cisR), and one non-cancerous fibroblast cell line (MRC-5) (Kostrhunova et al., 2023). R,R-1,2 cyclohexane diaminepyrophosphato-platinum (II) (PT-112) is another promising platinum-based compound (Kepp and Kroemer, 2020). Based on platinum (II) and pyrophosphate, PT-112 confers strong and long-lasting protective effects on murine colorectal carcinoma CT26 and MC38 cells making it a suitable inducer (Yamazaki et al., 2020). A phase I trial demonstrated its safety in advanced solid tumors (Karp et al., 2022). Moreover, the antimetabolite agent trifluridine/tipiracil may delete type-2 tumor-associated macrophages (a type of immunosuppressive cell) to modulate the TME in various microsatellite stable colon carcinoma cell lines (CT26, SW620, Caco-2, and Colo-320) (Limagne et al., 2019).

An increasing number of drugs are found to perform their antitumor role through ICD. For example, chromomycin A5 (CA5) can serve as a *bona fide* ICD inducer, since it enhances the activation of APCs and T-cells in syngeneic mouse models vaccinated with metastatic melanoma B16-F10 cells (Florêncio et al., 2022). Additionally, pemetrexed is a multi-targeting antifolate antagonist established as the first-line chemotherapeutic drug for advanced lung adenocarcinoma, and mesothelioma which increases immune-regulatory genes and induces ICD (Mathew et al., 2018; Sumiyoshi et al., 2021).

#### 3.1.2 Chemotherapy with nanotechnology

The application of nanotechnology has enhanced the strength of original drugs. Researchers organically assembled platinum (II) with novel norcantharidin co-delivery nanoparticles, and the dual synergistic antitumor activities of these nanoparticles were determined using an orthotopic 4T1 murine breast tumor model. These results strongly validated a promising nanotechnology delivering a chemo-immunotherapy paradigm for breast cancer treatment (Xiang et al., 2023). The same was true for other ICD-inducing agents. For example, DOX is a well-established ICD inducer that mediates immune responses through ROS, while ROS produced by DOX can be cleared by the redox functions of intracellular glutathione’s (GSH) (Ponsero et al., 2017). Jeon et al. (2023) encapsulated DOX into the core of a self-immolating polymer. These nanoparticles decomposed in the presence of ROS and formed a structure that strongly reacted with GSH, which effectively solved the problem of DOX-induced ROS-mediated immunogenicity decline caused by the GSH clearance of ROS in breast tumor cells (Jeon et al., 2023). Similarly, Hu et al. (2023) improved the tumor-resisting ability of DOX by depleting GSH in breast tumor cells. The difference is that the outer components of this nanocage can react directly with GSH without the need for other molecules to activate the structural transformation. In another study that combined aluminum hydroxide with DOX and tumor fragments, an improved nanovaccine showed a higher immune response and excellent inhibition of breast tumor growth (Tan et al., 2022). Nanotechnology is frequently used to combine two or more therapies to achieve synergistic effects. Researchers used nanotechnology to assemble DOX with near-infrared dyes to combine chemotherapy and photo-thermal therapy. This combination method significantly prolongs the median survival of breast cancer mouse models when compared to immunotherapy and ICD inducer alone, suggesting that this nanoparticle enhances antitumor immunotherapy (Zhu et al., 2023). Therefore, they provide further nanotechnology application references for combination therapies to improve immunotherapy. Other studies show that nanotechnology delivery systems with chemotherapy drugs may convert local immune tolerance (Zhang J. et al., 2021) and reduce systemic side effects (Moon et al., 2022).

### 3.2 Targeted therapy and small-molecule inhibitors (SMIs)

In recent years, further understanding of the ICD has stimulated researchers to discover and enhance the ICD-inducing role of targeted therapies. Increasing evidence suggests that many targeted agents exert their antitumor effects by inducing ICD. For example, osimertinib, an SMI known as an epidermal growth factor receptor (EGFR) tyrosine kinase inhibitor (TKI) demonstrates the potential to induce ICD in non-small cell lung cancer (NSCLC) tumor cells through the exposure and release of CRT (Furukawa et al., 2021). Lotsberg et al. (2020) reported that EGFR-TKI erlotinib-resistant cells can exhibit increased autophagic vacuolation and upregulation of the Anexelekto (AXL) receptor tyrosine kinase, whereas bemcentinib (which targets the AXL signaling pathway) can inhibit the transcription of genes associated with autophagy, release DAMPs, and trigger ICD in NSCLC cells. BI2536 is a selective PLK1 inhibitor that induces ICD to promote DC maturation and T-cell infiltration, thereby inducing apoptosis and altering the TME in NSCLC (Zhou et al., 2021). A mitogen-activated protein inhibitor drug, trametinib, could be effective in treating KRAS-mutant lung adenocarcinoma when combined with interleukin-12 by giving rise to ICD through sensitizing cancer cells to ERS and triggering the release of DAMPs (Wang et al., 2020). The EGFR-targeting antibody cetuximab, Bruton’s TKI ibrutinib, anaplastic lymphoma kinase TKI (crizotinib and ceritinib), mesenchymal-epithelial transition factor TKI foretinib, receptor protein TKI lestaurtinib, EGFR-TKI (lapatinib and canertinib), and CDK4/CDK6 inhibitors (abemaciclib and palbociclib) have also been demonstrated to present ICD activity before (Galluzzi et al., 2015; Inoue et al., 2017; Pozzi et al., 2016; Sagiv-Barfi et al., 2015; Schoenwaelder et al., 2021; Xu et al., 2022) (Table 1). Therefore, they have not been described in detail in this review.

There have also been many preclinical studies on SMIs and ICD in recent years. For example, CBL0137 (curaxin) targets histone chaperone FAcilitates Chromatin Transcription (FACT) to mainly interfere with the P53 and nuclear factor-κB (NF-κB) signaling pathways. Diffuse pleural mesothelioma cell lines treated with this SMI had a higher proportion of apoptotic cells and common cell cycle arrest compared to that seen in the control group. Simultaneously, high surface expression of important receptor signals and ICD markers during the long immune response were also observed (Singh et al., 2023). Waad et al. (2023) compared the ability of different SMIs to initiate type I IFN responses against multiple myeloma and identified PR619 (a proteasome inhibitor) as a novel ICD inducer.

The proposal of the ICD concept has also stimulated investigators to focus on the design of new targeted drugs to induce ICD. For example, researchers applied a CDK12/13 inhibitor (SR-4835) to mice with established 4T1 cells, a syngeneic mouse model of breast cancer, and found that SR-4835 can activate ERS, thus enhancing ICD. They also demonstrated that combining SR-4835 with anti-PD-1 can significantly inhibit tumor growth, providing a potential combination therapy for breast cancer (Li et al., 2020). Hwang et al. (2022) designed a phospholipase D (PLD) inhibitor to induce ICD to regulate TME in colorectal cancer, expecting to offer a promising strategy through this target drug. Moreover, researchers developed a novel fluorinated mitochondria-disrupting helical polypeptide to elicit mitochondrial dysfunction in murine colon adenocarcinoma CT26 cells. This resulted in ERS-mediated ICD, and suggested a synergistic effect with immunotherapy to reduce tumor cells numbers (Jeong et al., 2021). Lu et al. (2021) synthesized an activator (NBS-1MT) with a small molecule inhibitor and photosensitizer and found that it can induce oxidative stress to generate considerable ICD via pyroptosis in breast cancer cells. Although preclinical studies have laid a certain theoretical foundation, the clinical transformation of the combination of targeted therapy and ICIs is still not optimistic (Kroemer et al., 2023).

Taken together, targeted therapy exerts antitumor effects by interfering with various key steps in tumor formation. Although it has been investigated for decades, it still offers novel and innovative methods to improve antitumor efficacy. Identifying the most important targeting sites that can induce ICD is crucial to induce systemic immunity.

### 3.3 Antibody-drug conjugates (ADCs)

ADCs are new products that evolved during the process of targeted drug development (Hasan et al., 2018). ADCs are efficient drug delivery systems that combine targeted antibodies and cytotoxic antitumor drugs through linker molecules to achieve precise antitumoral effects. Like traditional targeted drugs, ADCs can activate ICD-related stress responses or signaling pathways. Heiser et al. (2023) applied Brentuximab Vedotin (an ADC targeting CD30) to the CD30-expressing lymphomas cell lines L540, HDLM-2, and A20, and tracking CRT provided evidence for the drug’s ICD-inducing properties. Mechanistically, the drug potently disrupts microtubule stability and leads to ERS, thereby activating ICD-related signaling pathways and immune cells (Heiser et al., 2023). PD-1 inhibition was subsequently combined to demonstrate the complementarity of the two approaches (Heiser et al., 2023). Similarly, the immunogenicity-inducing effects of another ADC drug targeting aberrantly expressed oncoproteins GPC2 (D3-GPC2-PBD) were explored. When applied to GPC2-expressing murine neuroblastomas, the drug caused changes in ICD markers such as CRT and HSP70/90, modulated the microenvironment, and improved macrophage phagocytosis, which was enhanced when CD47 was blocked by the combination (Pascual-Pasto et al., 2022). These examples suggest that ADCs are powerful ICD inducers with a promising future in combination therapies.

### 3.4 Radiotherapy

#### 3.4.1 Limitation of traditional radiotherapy in ICD

RT has been shown to elicit ICD, a typical manifestation of which is abscopal effect (Galassi et al., 2023; Hannon et al., 2023) (Table 1). Abscopal effect refers to an immune-related phenomenon in metastatic tumors, irradiating the orthotopic tumor leads to the inhibitory effect on non-orthotopic tumor cells. However, traditional RT-induced systemic immune responses are rare and insufficient to meet clinical needs (Huang et al., 2021). First, cells undergoing RT can lead to an increased proportion of regulatory T lymphocytes in tumor-infiltrating lymphocytes (Manukian et al., 2021), still making the microenvironment with “cold” characteristics. Second, some small molecules can cause radioresistance. For example, in the pancreatic cancer model, myeloid MyD88 (a downstream TLR expressed by macrophages and other immune cells) disturbs the production of Type I IFN, which is essential for T-cell activation, thus restricting the immune response to RT (Medler et al., 2023). Sun et al. (2022) demonstrated that glioblastoma multiforme cells receiving RT depend on the STAT1-IRF1-CD39 axis to upregulate the expression of CD39 to contribute to radioresistance. In addition to these suppressive factors in the TME, hypoxia and insufficient irradiation absorption are major mechanisms of resistance. Radiation dose and quality are closely related to the effectiveness of the immune response. Therefore, it is essential to determine the modality of RT, the dose regimen, and how to combine other methods to improve the efficacy of RT-induced ICD.

#### 3.4.2 Radiotherapy modalities

At present, heavy iron radiotherapy is an advanced RT method involving carbon ion and proton radiation that was successfully applied to cancer treatment. Compared to traditional photon therapy, heavy iron radiotherapy has stronger ICD potential and clinical application advantages. Carbon iron radiotherapy is an emerging type of ionizing radiation based on ^12^C^6+^. In addition to changes in ATP and the phosphorylation level of eIF2α, it leads to higher intranuclear HMGB1 efflux and mRNA level interferon expression in melanoma-bearing mice models compared with X-ray irradiation (Zhou et al., 2022). Proton radiation also presents a stronger tumor-killing effect on multiple human tumor cell lines (such as lung adenocarcinoma, glioma, tongue squamous carcinoma, and nasopharyngeal carcinoma), and has an equal effect to photon rays in inducing CRT exposure (Huang et al., 2019).

#### 3.4.3 Radiation dose fractionated technique

Technologies such as stereotactic body radiotherapy (SBRT) and spatially fractionated radiotherapy (SFRT) have made RT more efficient and can induce more ICD. SBRT has high precision and targets tumors with a very high radiation dose. It shows that tumor tissues in pancreatic ductal adenocarcinoma treated with SBRT have positive outcomes in terms of tumor density and ICD induction, although it does not lead to a decrease in immunosuppressive cells (Mills et al., 2022). To reduce the risk of damage to surrounding healthy tissues, SFRT was also introduced for use in clinical practice. It can deliver relatively high but different radiation doses over different fractions. It is distinguished by its steep dose gradient, which enables irradiation of the central hypoxic area of the tumor at high doses while preserving the function of surrounding tissues to elicit potent ICD (Massaccesi et al., 2020).

#### 3.4.4 Radiotherapy sensitizers

Several potential RT sensitizers have been identified to boost RT-induced ICD. For example, some compounds like Mn-based radiosensitizers ^131^I-MnO_2_-BSA mediate Fenton-like reactions to arrest the cell cycle and increase cell sensitivity to RT by regulating the cell cycle. Concurrently, it promotes the decomposition of hydrogen peroxide into O_2_ in breast and colon cancer model, thereby improving the resistance caused by the hypoxic environment (Zhang et al., 2022). Notably, improving the body’s ability to “correct errors” can also have a surprising effect. ATR inhibitors can inactivate serine/threonine protein kinases related to DNA repair, causing human lung cancer and osteosarcoma cells that fail to repair DNA to undergo mitotic death (Eek et al., 2023). Similarly, ER-associated protein degradation inhibitors can interfere with key proteins in the degradation of misfolded proteins, which is conducive to the amplification of mild ERS induced by RT in esophageal squamous cell carcinoma cell lines (Luo H. et al., 2023). In addition, some sensitizers can improve the efficacy of RT by enhancing key events in the RT-induced ICD process, such as inducing intracellular DNA damage (Zhuang et al., 2023), triggering ferroptosis (Luo et al., 2022), and activating ERS PERK-eIF2α and IRE1α-XBP1 signaling pathways (Xiu et al., 2022; Yang et al., 2020).

#### 3.4.5 Nanotechnology radiotherapy

Nanotechnology has unique advantages when used to improve the efficacy of RT, such as achieving precise RT positioning. In experiments using murine melanoma B16 cells, Song et al. (2022) developed a gadolinium-based, highly efficient, guided irradiation nanoparticle to exacerbate radiation-induced DNA damage, cell cycle arrest, and ICD. In addition to improving immune function, the excellent radiation deposition and tumor penetration ability of nanomaterials were exploited to overcome the problem of low radiation absorption in tumor tissues (Janic et al., 2021; Wang X. et al., 2022). Gold nanoparticles (AuNPs) have promising radiosensitizing effects and can enhance RT-induced ICD in glioblastoma (He C. et al., 2023). Furthermore, the application of nanotechnology can result in an optimal combination of multiple drugs and therapies. Cisplatin enhances radiation-induced abscopal effects in melanoma, colon carcinoma and breast cancer (Luo R. et al., 2023). Cisplatin-based nanoparticles have a greater ability to enhance T cell infiltration and abscopal effects in Lewis lung carcinoma than cisplatin alone (Wang X. et al., 2021). Thus, a synergistic effect was achieved between chemotherapy and RT. Simultaneously, immunotherapy can also be used to generate a stronger antitumor effect (Choi et al., 2020; Song et al., 2022).

### 3.5 Photodynamic therapy

PDT is a treatment wherein the tumor tissue is dosed with a photosensitizer and locally irradiated with light of an appropriate wavelength to activate the photosensitizer and produce ROS that exert cytotoxic effects. Sun and collaborators demonstrated that the mechanism by which the photosensitizer B5-aminolevulinic acid (5-ALA) exerts antitumoral effects in mouse models of colorectal cancer, is achieved by ICD induction, depending on protein kinase B (AKT) inhibition and effective activation of bone-marrow derived dendritic cells (Sun et al., 2023). Thus, PDT can serve as a *bona fide* ICD inducer to inhibit tumor progression. Notably, different photosensitizer types, concentrations, and light doses can affect the PDT efficacy (Lin et al., 2022; Rodrigues et al., 2022).

Numerous issues are receiving considerable attention along the process. First, the body has a repair system for the DNA damage caused by ROS, which weakens the immune function of photosensitizers. To solve this problem in breast cancer, researchers have used poly (ADP-ribose) polymerase 1 (PARP1) inhibitors to disrupt the repair function and enhance the antitumor activity of photosensitizers (Li et al., 2023). The second problem that needs to be urgently solved is hypoxia and immunosuppression of the microenvironment caused by tumors, which are not conducive to ROS production. Wang et al. (2023) used nanotechnology to combine photosensitizers, the hypoxia-activated prodrug tirapazamine, and immunosuppressants. Oxygen-boosted PDT was also developed to overcome this limitation (Alzeibak et al., 2021). Moreover, targeting strategies can significantly improve the selectivity and efficacy of photodynamic agents in tumor tissues and have attracted extensive attention. Generally, these strategies can be divided into active and passive targets. Active targeting is associated with the structural characteristics of photodynamic agents that have a high affinity for tumor cells. Zeng et al. (2023) proposed a strategy for fabricating photosensitizers that precisely target the mitochondria, lysosomes, and ER in triple-negative breast cancer (TNBC) 4T1 cell lines to induce pyroptosis and selectively damage localized organelles based on cyanine chromophores and heavy atoms. Li J. et al. (2022) took advantage of the lipophilicity of phosphindole oxide to give full play to the aggregation-induced emission characteristics so that the novel photosensitizer could cross the cell membrane of cervical carcinoma HeLa cell and aggregate in the ER. However, passive targeting often involves the participation of nanomaterials, such as organic micelles (Liang et al., 2023), nano-metal frames (Zhang X. et al., 2021) and composite nanomaterials (Tian et al., 2023; Xiang et al., 2022).

### 3.6 Other potential inducers

#### 3.6.1 Isolated natural drugs and traditional Chinese medicine

Multiple natural drugs or traditional Chinese medicines can induce ICD and inhibit tumor growth. The application of γ-mangostin to a leukemia mouse model shows that the size and weight of the mouse spleen is significantly reduced after its treatment, while the survival time of the mouse is significantly extended, indicating its benefit in leukemia treatment (Long et al., 2023). Further investigation demonstrates that this effect could be achieved by inducing ICD and activating the cGAS pathway (Long et al., 2023). Lepadin A is another natural compound with ICD inducing activity. In experiments using human melanoma A2058 cells, lepadin A induced higher CRT exposure and translocation, which was closely related to a CD91-dependent pathway that triggered the maturation and activation of DCs (Carbone et al., 2023). Afzelin is a flavonol glycoside found in various plants that can inhibit lung cancer progression by targeting NQO2, thereby activating ERS and inducing ICD (Xia et al., 2023). Therefore, natural drugs may provide benefits to patients with tumors in an ICD-inducing manner.

Notably, the emergence of the ICD concept may help researchers recognize the potantial clinical usage of traditional Chinese medicine in oncology therapeutic areas. Researchers (Sha et al., 2022) found that honey-processed *Astragalus* had increased antitumor immune activity in NSCLC, colon carcinoma and melanoma models compared to that seen with unprocessed *Astragalus* and the mechanism may be related to the induction of apoptosis and immunogenicity. In addition, the extract of the traditional Chinese medicine *Marsdenia tenacissima* activates ERS and ICD via AXL suppression to treat NSCLC (Yuan et al., 2023). *Trametes robiniophila* Murr. (Huaier) stimulates ICD in TNBC cells by promoting CRT exposure and increasing the release of ATP and HMGB1. Furthermore, Huaier may play a role in promoting ICD through the circCLASP1/PKR/eIF2α axis signaling pathway *in vitro* and *in vivo* (Li Z. et al., 2022). Self-assembled traditional Chinese nanomedicine using ursolic acid and lentinan can expand the ICD and improve the efficacy of immunotherapy in colorectal cancer (Mao et al., 2022). Licoricidin can affect the ERS of cervical cancer cells and further the release of DAMPs, thus trigger the emergence of ICD (Wu et al., 2023).

#### 3.6.2 Microbiota therapy

It is widely acknowledged that microbiota plays an important role in mediating the efficacy of immunotherapy as it affects systemic immune responses. Investigators suggest that this effect (at least in part) is potentially mediated by ICD (Chen et al., 2020). With the help of nanotechnology, microbiota can be used as efficient carriers of antitumor drugs that can enhance the two-way killing effect of microbiota and drugs. *Fusobacterium nucleatum* can colonize TNBC and maintain the inhibitory properties of the TME. Based on this biological property, a nanomedicine was developed to precisely target and kill TNBC cells while also killing *F. nucleatum*, thereby regulating the immune microenvironment, which is conducive to turning “cold” tumors into “hot” tumors (Liu Z. et al., 2023). Moreover, researchers have used the anaerobic properties of *Bifidobacterium infantis*(B) as a loader for nanoparticles to selectively transport photosensitizers to the hypoxic region of colorectal cancer cells, providing a creative solution to solve the problems of tumor metastasis and drug resistance caused by hypoxia (Dai et al., 2023). *Salmonella* VNP20009 is a bacterial vector that plays the same role as the microbiota described above. Combining microbiota therapies with radiosensitizers achieves good biosafety while exhibiting an immune-friendly abscopal effect (Duo et al., 2023). The bacterial colonization of tumors also induces adaptive immune responses and exerts antitumor effects (Xie et al., 2023). Nanoparticles composed of *Bifidobacteria* and DOX, strategically combined bacterial therapy with chemotherapy, significantly inhibited melanoma tumor progression (He T. et al., 2023).

#### 3.6.3 Tumor-treating fields (TTFields)

TTFields are new portable, non-invasive, physical anticancer therapies that interfere with tumor cell mitosis by acting on tubulin using a low-intensity, medium-frequency alternating current electric field. An increasing number of studies have focused on the potential effect of TTFields in eliciting ICD and the synergistic efficacy of TTFields with immunotherapy with the expectation of achieving better tumor control. For example, Voloshin et al. (2020) demonstrated that TTFields effectively potentiated ICD, and elicited a potent adaptive immune response through upregulation of DAMPs, including HMGB1, ATP, and CRT. Moreover, integrating TTFields with anti-PD-1 could enhance antitumoral immunity and achieve better tumor control in the murine colon CT-26 tumor model. Coincidentally, syngeneic NSCLC mouse models demonstrated that the presence of ICD *in vivo* during TTFields therapy concomitant with immunotherapy could alter the immune microenvironment and increase immune activity (Barsheshet et al., 2022). Moreover, the overall survival of TTFields plus standard therapy (including nivolumab, pembrolizumab, atezolizumab, and docetaxel) was significantly superior to that of standard therapy alone in a randomized phase 3 clinical trial of 276 patients with metastatic NSCLC who had been previously treated with platinum. In this context, TTFields is an alternative treatment for metastatic NSCLC that progresses with platinum-based drugs (Leal et al., 2023). Perhaps, the combination of TTFields and other therapeutic regimens will have broad application prospects in the treatment of many types of tumors.

Overall, these therapeutics mentioned above are still far from sufficient for clinical transformation. Therefore, *in vivo* experiments and clinical trials are urgently needed to exploit more potential *bona fide* ICD inducer candidates, combine existing diverse regimens, or design more novel polymers to meet clinical needs.

## 4 Clinical application

We summarized some of the completed trials published on the ClinicalTrials.gov database in recent years on ICD inducers in combination with immunotherapy from the ClinicalTrials.gov database in recent years, including chemical agents, novel RT, and target therapies (Table 2). Among them, eight studies related to chemotherapy with immunotherapy, seven studies related to radiotherapy with immunotherapy and three studies related to targeted therapy with immunotherapy. Multiple cancer types were included in these trials and a large portion of them focused on TNBC and lung cancer, while others included cervical cancer, uterine cancer, head and neck cancer, melanoma and lymphoma. Additionally, there were two “basket trials” consisting of multiple solid tumors. The role of ICD in clinical application is important but remains difficult to evaluate owing to limited induction protocols, inaccurate monitoring indicators, and limited access to samples (Fabian et al., 2022; Rapoport and Anderson, 2019). Most previous prospective clinical trials have used ICD as an exploratory endpoint and supported the beneficial effects of ICD in the prognosis of some patients with cancer (Fu et al., 2022; Wang Y. et al., 2022). Most of our summarized studies also evaluate ICD as an exploratory endpoint by the measurements of immunological parameters or investigated immune responses. From these studies, the effect of ICD is usually indirectly manifested through DAMPs or immune biomarkers (including, but not limited to, PD-L1 expression levels, T-cell density, and cytokine levels). Some of these trials confirmed the association of ICD with better clinical outcomes. Also, most of the summarized trials focus on combination regimens. For example, a clinical trail (NCT02622074) explored the antitumor activity of immunomodulatory chemotherapy regimens plus pembrolizumab in 60 patients with early-stage TNBC, finding a positive correlation between biomarker levels and clinical outcomes (Schmid et al., 2020). In another phase II randomized controlled trial (NCT03164993), to investigate the efficiency of adding immunotherapy to ICD inducers, researchers combined DOX with cyclophosphamide and atezolizumab to treat 68 patients with TNBC. Flow cytometry data showed that Treg counts reduced after low doses of metronomic cyclophosphamide, thus providing favorable evidence for the hypothesis that the semi-metronomic chemotherapy regimen sensitize patients to immunotherapy through ICD inducing and immunosuppressive cells reducing (Kyte et al., 2020; Røssevold et al., 2022). The trail (NCT02492568) enrolled 92 patients with advanced NSCLC, and the results showed that the objective response rate value was 36% in patients who received SBRT plus pembrolizumab versus 18% in patients who applied pembrolizumab alone. Overall, different combinations of ICD inducers and immunotherapies could potentially benefit patients through the ICD effect. These results emphasize the hopeful prospects and clinical directions in ICD research.

**TABLE 2:** Completed clinical trials related to ICD inducers combined with immunotherapy.

Trail number	Cancer type	Interventions	Phase
Chemotherapy			
NCT01637532	Ovarian Cancer	Carboplatin, caelyx/doxorubicin, tocilizumab and interferon alpha 2-b	Ⅰ/Ⅱ
NCT02622074	TNBC	Nab-paclitaxel, doxorubicin, cyclophosphamide, carboplatin, paclitaxel and pembrolizumab	Ⅰ
NCT02819518	TNBC	Nab-paclitaxel/paclitaxel/gemcitabine/carboplatin and pembrolizumab	Ⅲ
NCT03003520	High-risk diffuse large B-cell lymphoma	Rituximab, doxorubicin, vincristine, cyclophosphamide, prednison/lenalidomide and durvalumab	Ⅱ
NCT03117049	Non-squamous NSCLC	Carboplatin, paclitaxel, bevacizumab and nivolumab	Ⅲ
NCT03164993	TNBC	Pegylated liposomal doxorubicin, cyclophosphamide and atezolizumab	Ⅱ
NCT03289819	TNBC	Cyclophosphamide, nab-paclitaxel, epirubicin and pembrolizumab	Ⅱ
NCT03432598	Locally Advanced/Metastatic Lung Cancer	Paclitaxel, gemcitabine, etoposide, pemetrexed, cisplatin, carboplatin and tislelizumab	Ⅱ
Targeted therapy			
NCT02764593	Advanced Head and Neck Cancer	Cetuximab, RT and nivolumab	Ⅰ
NCT02403271	Solid Tumors	Ibrutinib and durvalumab	Ⅰ/Ⅱ
NCT00684983	Breast cancer	Lapatinib ditosylate, capecitabine and cixutumumab	Ⅱ
Radiotherapy			
NCT01711515	Cervical cancer	RT, cisplatin and IPI	Ⅰ
NCT02221739	NSCLC	RT and IPI	Ⅰ/Ⅱ
NCT02239900	Advanced solid tumors	SBRT and IPI	Ⅰ
NCT02406183	Melanoma	SBRT and IPI	Ⅰ
NCT02492568	Advanced NSCLC	SBRT and pembrolizumab	Ⅱ
NCT02904954	NSCLC	SBRT and durvalumab	Ⅱ
NCT03192059	Cervical/Uterine cancer	Pembrolizumab, RT and modulatory cocktail	Ⅱ

NSCLC, non-small cell lung cancer; TNBC, triple-negative breast cancer; RT, radiotherapy; SBRT, stereotactic body radiotherapy; IPI, ipilimumab.

We also assessed clinical studies of ICD inducers in combination with immunotherapy currently underway some of which are presented in Table 3. Among them, eight studies related to chemotherapy with immunotherapy, nine studies related to radiotherapy with immunotherapy and 9 studies related to target therapy with immunotherapy. Unlike the completed studies described above, the ongoing clinical trials are predominately focused on colorectal cancer. Most trials of chemotherapy combined with immunotherapy use oxaliplatin as an interventional method, which is recognized as one of the most effective chemotherapy drugs for colorectal cancer as well as an effective ICD inducer. With respect to induction of ICD by novel radiation therapy, SBRT with ICIs was assessed in four trials, and there were two trials assessed heavy ion radiotherapy with ICIs. In these trials, multiple immunological markers were examined to predict the efficacy of immunotherapy during treatment.

**TABLE 3:** Clinical trials ongoing related to ICD inducers combined with immunotherapy.

Trail number	Cancer type	Interventions	Phase
Chemotherapy			
NCT03388190	Colorectal cancer	OXA+5-FU + NIVO	Ⅱ
NCT04043195	Advanced NSCLC	OXA + NIVO + IPI	Ⅰ/Ⅱ
NCT04159818	TNBC	DOX + DDP + NIVO	Ⅱ
NCT04262687	Colorectal cancer	OXA + CAP + BEV + Pembrolizumab	Ⅱ
NCT05307198	Rectal neoplasms	OXA + CAP + Sintilimab	Ⅱ
NCT05420584	Rectal neoplasms	OXA + CAP + Tislelizumab	Ⅱ
NCT05504252	Colorectal cancer	OXA + NIVO	Ⅱ
NCT05144698	Solid tumor	PTX + CBP + RAPA-201	Ⅰ/Ⅱ
Targeted therapy			
NCT05019534	Colotectal cancer	Cetuximab + Vemurafenib + Camrelizumab	Ⅰ
NCT03666325	Cutaneous squamous cell cancer	Cetuximab + Pembrolizumab	Ⅱ
NCT03608046	Metastatic colorectal cancer	Cetuximab + Avelumab + Irinotecan	Ⅱ
NCT05260671	Head and neck cancer	Cetuximab + penpulimab	Ⅱ
NCT04745130	Advanced colorectal cancer	Cetuximab + Sintilimab + Regofinib	Ⅱ
NCT04751929	Prostate cancer	Abemaciclib + Atezolizumab	Ⅱ
NCT06113809	Undifferentiated pleomorphic sarcoma	Palbociclib + Pembrolizumab	Ⅰ
NCT03021460	Melanoma	Ibrutinib + Pembrolizumab	Ⅰ
NCT04017650	Colorectal cancer	Cetuximab + Encorafenib + NIVO	Ⅰ/Ⅱ
Radiotherapy			
NCT06133062	Hepatocellular carcinoma	Proton radiotherapy + Bevacizumab + Atezolizumab	Ⅱ
NCT05229614	NSCLC, Head and neck cancer, Melanoma, Urothelial carcinoma	Carbon ion radiotherapy + Pembrolizumab	Ⅱ
NCT03223155	NSCLC	SBRT + NIVO + IPI	Ⅰ
NCT05401786	Advanced NSCLC	SBRT + IPI + Cemiplimab	Ⅱ
NCT06074692	Sarcoma	SBRT + Camrelizumab + Fluzoparib	Ⅱ
NCT06114225	Osteosarcoma	SBRT + Gemcitabine + Penpulimab	Ⅱ
NCT02888743	Colorectal cancer, NSCLC	RT + Durvalumab + Tremelimumab	Ⅱ
NCT03104439	Colorectal/Pancreatic cancer	RT + NIVO + IPI	Ⅱ
NCT05445648	Urinary bladder neoplasms	RT + Tislelizumab	Ⅱ

DOX, doxorubicin; EPB, epirubicin; MIT, mitoxantrone; IDA, idarubicin; DDP, cisplatin; OXA, oxaliplatin; PTX, paclitaxel; DTX, docetaxel; CAP, capecitabine; NIVO, nivolumab; IPI, ipilimumab; NSCLC, non-small cell lung cancer; TNBC, triple-negative breast cancer; MSS, microsatellite stable; SBRT, stereotactic body radiotherapy; RT, radiotherapy.

## 5 Future development and perspectives

It is worth noting that different ICD inducers have their drawbacks. For example, chemotherapy has a risk of severe systemic side effects and drug resistance, while targeted therapy is highly selective. Although RT as a local treatment has relatively few side effects and can confer an abscopal effect on tumor cells at the irradiation site, it leads to an increased proportion of immunosuppressive cells. Thus, it is challenging to regulate its impact on the immune system. PDT has limited penetration and is only suitable for superficial tumors. To some extent, new technologies, such as nanotechnology, compensate for the inherent defects of these ICD inducers by enhancing the stability and efficacy of drugs, improving the precision of action, reducing side effects, and enabling the combination of multiple treatment methods. However, there are still some limitations that must be addressed. First, it is difficult to identify the patients who will benefit the most and those who will not benefit, although researchers have proposed various prognosis models for patient stratification, such as ICD-related gene expression level (Jiang et al., 2023; Wang Y. et al., 2021; Zhang et al., 2023). Based on the biological mechanisms of ICD, we hypothesize that patients with low immunogenic malignancies are less likely to benefit from it. In addition, an increased number of side effects may occur when multiple approaches are used simultaneously to manage tumors or when applied to older or frail individuals. Second, the development of DAMPs has had a disproportionate focus on established markers, and there are few novel markers. Third, most therapeutics do not consider ICD activation as their main mechanism of action, and it is not easy to compare the efficiency of ICD eliciting ability. Finally, limited attention has been paid to the induction protocol, the timing and the doses of the inducer. However, we believe that these current obstacles will be appropriately resolved shortly. With technological advances, the detection and clinical efficacy of ICD could be enhanced to better understand the potential role of ICD induction in combination with immunotherapy. Appropriate combination of regimens with ICD inducers with immunotherapy for different tumors will be developed, and a therapeutic strategy for immunosuppressive tumors will be formulated to better treat distal tumors and maintain memory immunity, allowing wider use of personalized treatment.

## 6 Conclusion

Although the clinical application of immunotherapy has prolonged the survival of patients with cancer to a considerable extent, the treatment of malignant tumors remains a major problem in medicine today. ICD induction has emerged as an effective enhancement method to address the limited use of immunotherapy for “cold” tumors, and it is expected to further improve patient outcomes. The process is often accompanied by antigenicity, the secretion of DAMPs, and the activation of conduction pathways to achieve alterations in the TME and systemic immune responses. As a feasible therapeutic strategy in antitumor therapy, further elucidating the mechanism of ICD and developing new combination of regimens will benefit an increasing number of patients with cancer.

## Figures and Tables

**Table:** 

**TME**	tumor microenvironment
**ICD**	immunogenic cell death
**DAMPs**	damage associated molecular patterns
**RT**	radiotherapy
**PDT**	photodynamic therapy
**DCs**	dendritic cells
**TNAs**	tumor neoantigens
**TAAs**	tumor-associated antigens
**ERS**	endoplasmic reticulum stress
**ROS**	reactive oxygen species
**CRT**	calreticulin
**HMGB1**	high-mobility group protein box 1
**ATP**	adenosine triphosphate
**HSP**	heat shock proteins
**PGE2**	prostaglandin E2
**ADO**	adenosine
**ER**	endoplasmic reticulum
**PT-112**	R,R-1,2 cyclohexane diaminepyrophosphato-platinum (II)
**CA5**	chromomycin A5
**DOX**	doxorubicin
**EPB**	epirubicin
**MIT**	mitoxantrone
**IDA**	idarubicin
**DDP**	cisplatin
**OXA**	oxaliplatin
**PTX**	paclitaxel
**DTX**	docetaxel
**CAP**	capecitabine
**NIV**	Onivolumab
**IPI**	ipilimumab
**ICIs**	immune checkpoint inhibitors
**GSH**	glutathione
**SMIs**	small-molecule inhibitors
**TKI**	tyrosine kinase inhibitor
**NSCLC**	non-small cell lung cancer
**FACT**	FAcilitates Chromatin Transcription
**NF-κB**	nuclear factor-κB
**PLD**	phospholipase D
**ADCs**	antibody-drug conjugates
**SBRT**	stereotactic body radiotherapy
**SFRT**	spatially fractionated radiotherapy
**AuNPs**	Gold nanoparticles
**PARP1**	poly (ADP-ribose) polymerase 1
**TNBC**	triple-negative breast cancer
**TTFields**	tumor-treating fields
**TLR-4**	Toll-like receptor 4
**IRE1α**	inositol-requiring enzyme 1 alpha
**XBP1**	X-box-binding protein 1
**PERK**	protein kinase RNA-like endoplasmic reticulum kinase
**eIF2α**	eukaryotic initiation factor-2α
**ATF4**	activating transcription factor 4
**ATF6**	activating transcription factor 6
**IFN**	interferon
**ERp57**	protein disulfide isomerase A3
**EGFR**	epidermal growth factor receptor
**CDK**	cyclin-dependent kinase
**MHC-1**	major histocompatibility complex class 1
**MSS**	microsatellite stable
**AXL**	Anexelekto
